# Atomic Simulation of the Binding of JAK1 and JAK2 with the Selective Inhibitor Ruxolitinib

**DOI:** 10.3390/ijms231810466

**Published:** 2022-09-09

**Authors:** Maxim Kondratyev, Vladimir R. Rudnev, Kirill S. Nikolsky, Alexander A. Stepanov, Denis V. Petrovsky, Liudmila I. Kulikova, Arthur T. Kopylov, Kristina A. Malsagova, Anna L. Kaysheva

**Affiliations:** 1 Institute of Cell Biophysics, Russian Academy of Sciences, 142290 Pushchino, Russia; 2 Biobanking Group, Branch of Institute of Biomedical Chemistry “Scientific and Education Center”, 109028 Moscow, Russia; 3 Institute of Theoretical and Experimental Biophysics, Russian Academy of Sciences, 142290 Pushchino, Russia; 4 Institute of Mathematical Problems of Biology RAS—The Branch of Keldysh Institute of Applied Mathematics of Russian Academy of Sciences, 142290 Pushchino, Russia

**Keywords:** ruxolitinib, JAK inhibitor, rheumatoid arthritis, molecular modeling

## Abstract

Rheumatoid arthritis belongs to the group of chronic systemic autoimmune diseases characterized by the development of destructive synovitis and extra-articular manifestations. Cytokines regulate a wide range of inflammatory processes involved in the pathogenesis of rheumatoid arthritis and contribute to the induction of autoimmunity and chronic inflammation. Janus-associated kinase (JAK) and signal transducer and activator of transcription (STAT) proteins mediate cell signaling from cytokine receptors, and are involved in the pathogenesis of autoimmune and inflammatory diseases. Targeted small-molecule drugs that inhibit the functional activity of JAK proteins are used in clinical practice for the treatment of rheumatoid arthritis. In our study, we modeled the interactions of the small-molecule drug ruxolitinib with JAK1 and JAK2 isoforms and determined the binding selectivity using molecular docking. Molecular modeling data show that ruxolitinib selectively binds the JAK1 and JAK2 isoforms with a binding affinity of −8.3 and −8.0 kcal/mol, respectively. The stabilization of ligands in the cavity of kinases occurs primarily through hydrophobic interactions. The amino acid residues of the protein globules of kinases that are responsible for the correct positioning of the drug ruxolitinib and its retention have been determined.

## 1. Introduction

Rheumatoid arthritis (RA) can lead to severe disability; therefore, timely and effective treatment is important to reduce the negative effects of functional damage (deformation and destruction) to the joints, which occurs at an early stage of RA development [1]. There are no specific RA serological markers that allow a high-precision diagnosis of RA at an early stage before the onset of clinical symptoms in current clinical practice [2]. A deep study of the molecular basis of the disease’s development seems to be relevant to the development of new approaches to RA prevention and cure. The most attractive approaches are non-therapeutic ones (physical activity, diet) that, on the one hand, improve the general state of human health, and, on the other hand, reduce the negative symptoms of RA development (inflammation, pain, joint damage). The literature presents the results of studies that confirm the positive effect of long-term, high intensity exercise on the general health of patients with rheumatoid arthritis and knee osteoarthritis [3,4,5,6]. The authors of the studies agree that the deterioration in health status in RA is exacerbated by the avoidance of exercise [5,6]. The authors of a number of studies note cases of combined progression in patients with RA and intolerance to certain food allergens [2,7,8,9]. Studies show a positive effect of short-term fasting, a vegan diet, and an elimination diet on improving the health of RA patients, which is likely due to a reduction in immune reactivity to certain food antigens in the gastrointestinal tract that are eliminated by changing the diet [10,11].

**Figure 1:**
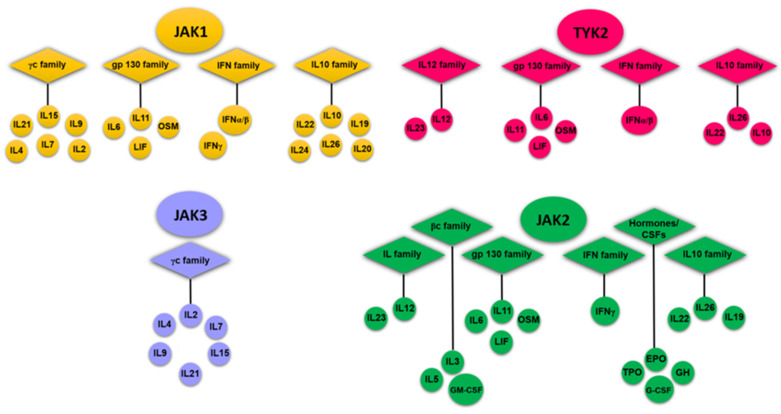
Cytokines that signal through the JAK family proteins. IL-6 signaling, which plays an important role in the pathogenesis of RA, leads to the activation of JAK1, JAK2, and TYK2. Erythropoietin receptor (EpoR) signaling is mediated by JAK2 and is important in the development and deployment of reticulocytes and erythrocytes. JAK3 in combination with JAK1 is an important signaling component for cytokine receptors that share a common gamma chain (γc), such as IL-2, IL-4, IL-7, IL-9, IL-15, and IL-21.

The activation of lymphocytes and synthesis of pro-inflammatory cytokines by macrophages, primarily interleukin 1 beta (IL1β) and tumor necrosis factor alpha (TNFα), play a key role in the inflammation of the synovial membrane in RA. These interleukins contribute to the persistence of the inflammatory process in the synovial membrane and the destruction of cartilage and bone tissue, because of the direct effect on synovial fibroblasts, chondrocytes, and osteoclasts. Similar to the inflammatory process, cyclooxygenase-2 (COX-2) is activated, leading to an increase in the synthesis of prostaglandins. Disease-modifying antirheumatic drugs (DMARDs), which are immunosuppressive and immunomodulatory agents, are currently widely used in the clinical treatment of RA [12]. Janus-associated kinase (JAK) inhibitors are a new class of targeted synthetic DMARDs (tsDMARDs) used to treat RA [13]. tsDMARDs provide targeted inhibition of JAKs, which plays a key role in the pathogenesis of RA [1]. JAK family enzymes are represented by four isoforms: JAK1, JAK2, JAK3, and tyrosine kinase 2 (TYK2), which are important signaling molecules involved in cytokine and growth factor signaling [14,15]. The cytokine receptors signal through the JAK–STAT pathway [15]. JAK molecules act together in cytokine signaling (the majority of cytokine receptors use three JAK combinations), but under certain conditions, they exhibit selectivity for one isoform (Figure 1).

Isoforms of the JAK family exhibit cross-activity. JAK1 plays an important role in the signaling of several pro-inflammatory cytokines, often in collaboration with other members of the JAK family. Thus, JAK2 is used mainly by receptors for hematopoietic growth factors, such as erythropoietin and thrombopoietin. JAK3 is thought to play a major role in the mediated activation of immune function, while TYK2 functions in association with JAK2 or JAK3 to signal cytokines such as interleukin-12 (IL-12) [16].

Timely and effective treatment of RA at an early stage is essential to control joint damage. Small molecule JAK inhibitors represent a new class of drugs for the treatment of rheumatoid arthritis.

Ruxolitinib (KEGG ID D09960) is a tsDMARD with the International Union of Pure and Applied Chemistry (IUPAC) name (3R)-3-cyclopentyl-3-[4-(7H-pyrrolo [2,3-d]pyrimidin-4-yl) pyrazol-1-yl] propanenitrile and a molecular weight of 306.4 Da [17]. Ruxolitinib is a pyrazole, substituted at position 1 by a 2-cyano-1-cyclopentylethyl group, and at position 3 by a pyrrolo [2,3-d]pyrimidin-4-yl group. Ruxolitinib (INCB018424) is the first potent and selective JAK1/2 inhibitor to be approved for medical use, with a half maximal inhibitory concentration (IC50) of 3.3 nM/2.8 nM in cell-free assays and >130-fold selectivity for JAK1/2 versus JAK3 [18].

Ruxolitinib is a class I molecule of the Biopharmaceutical Classification System (BCS) with high permeability, high solubility, and rapid disintegration. Ruxolitinib is primarily metabolized by the CYP3A4 cytochrome P450 family proteins (>50%) via CYP2C9. At clinically significant concentrations, the drug does not inhibit CYP1A2, CYP2B6, CYP2C8, CYP2C9, CYP2C19, CYP2D6, or CYP3A4, and is not a potent inducer of CYP1A2, CYP2B6, or CYP3A4. The drug is used in myeloproliferative neoplasms and autoimmune diseases, which are known to be associated with the dysregulation of JAK1 and JAK2. This dysregulation is believed to be due to the high levels of circulating cytokines, which are associated with JAK–STAT pathway activation, JAK2V617F mutations, and the down-regulation of negative regulatory mechanisms. Ruxolitinib inhibits JAK–STAT signaling and cell proliferation in cytokine-dependent cell models of hematologic malignancies, and Ba/F3 cells become cytokine-independent by the expression of JAK2V617F mutant protein, with IC50 ranging from 80 to 320 nM [19]. Ruxolitinib has two chiral forms: S2902 S-Ruxolitinib is the S form and S1378 Ruxolitinib is the D form. One of the carbons in this molecule is asymmetric, rendering the two molecules mirror images of each other. It is probable that the biological activities of these two molecules is different. Numerous studies show that ruxolitinib, a JAK2 inhibitor, suppresses proliferation and induces apoptosis of mutated JAK2V617F cell lines [19,20,21,22].

This study was performed to model the interactions of ruxolitinib with JAK1 and JAK2 isoforms, and to determine the binding selectivity using molecular docking.

## 2. Results

### 2.1. Characterization of the “Grotto” of Ligand Binding for JAK Isoforms

**Figure 2:**
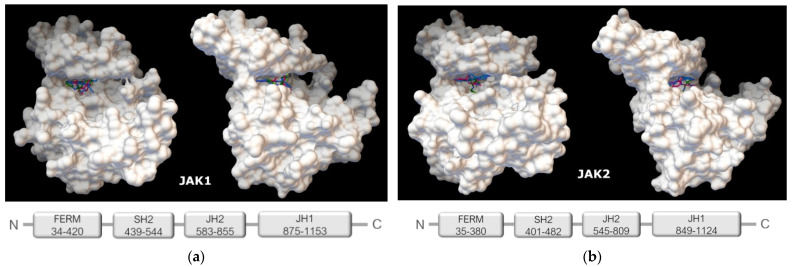
Topology of the “grotto” involved in the binding of low molecular weight ligands (view in two projections) in the globule of the JAK1 protein (locus 865-1154 a.a.) (**a**), JAK2 (locus 842-1130 a.a.) (**b**).

Calculations show that the studied proteins and ligands bind very specifically. The van der Waals surface of the globule of each protein has a niche, or “grotto”, in which each of the studied ligands is completely sterically placed. Externally, the topology of the binding proteins and ligands appears very similar in the “grotto” (Figure 2).

The “grotto” for the JAK1 isoform is formed by a fragment of the amino acid sequence 865–1154 of the JH1 domain, which exhibits tyrosine kinase activity [20]. The secondary structure of the “grotto” is formed by 13 β-strands and 17 α-helices (according to structural identification (STRIDE)), which form the fold of Scope ID d1t46a. The protein belongs to structural class d: alpha and beta proteins (a + b). The “grotto” for the JAK2 isoform is formed by a fragment of the amino acid sequence 842–1130, also of the JH1 domain. The secondary structure of the “grotto” is rich in β-strands (12) and contains 17 α-helices that form the fold of Scope ID d1u46a. This protein also belongs to structural class d: alpha and beta proteins (a + b).

The physicochemical and structural parameters of the “grotto” are similar for both the isoforms. Thus, the area accessible to the solvent “grotto” of JAK1 and JAK2 is 29,255.34 Å^2^ and 31,252.36 Å^2^, respectively (Table 1).

Interesting results were obtained from the analysis of possible contacts between amino acid residues involved in the formation and stabilization of the “grotto” structures. Indeed, we observe that in selected regions of amino acid sequences, approximately 40% of the sequence is made up of hydrophobic amino acid residues. An analysis of possible contacts between amino acid residues reveals a bimodal distribution of distances between the interacting hydrophobic amino acids. The number of contacts formed within one element of the secondary structure (inside-elements) is only a small fraction of probable contacts (Table 1, column “Nonpolar “inside-elements” contacts”), most of which can be attributed to the interactions of amino acid residues localized in different elements of the secondary structures (Table 1, column “Nonpolar “cross-elements” contacts”). Of note is the compactness of the structural motifs that form the “grottoes”, which is due to the presence of two maxima in the distribution of distances between the interacting hydrophobic amino acid residues. The first maximum characterizing contacts between amino acid residues is observed within 7.7–7.8 Å, and the second maximum for contacts between elements of secondary structures, is observed for distances of 8.3 Å.

### 2.2. Results of Molecular Docking

**Figure 3:**
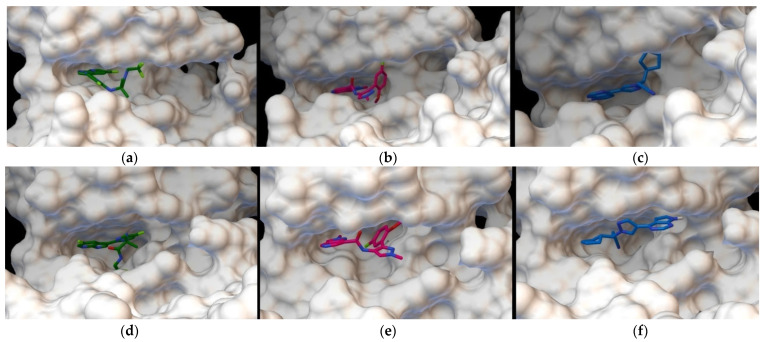
Ligand binding site and JAK1: (**a**) decernotinib, (**b**) KEV, and (**c**) ruxolitinib; ligands and JAK2: (**d**) decernotinib, (**e**) KEV, and (**f**) ruxolitinib (Supplementary Materials, Figures S1–S6).

Calculations of flexible docking make it possible to estimate the binding affinity, or “affinity”. This value is the total energy indicated in kcal/mol with a negative sign, as these complexes are stable. According to our data, the affinity energy of decernotinib (known as ligand-467 from the experimentally solved 4YTH complex in PDB) for the JAK1 protein is −8.7 kcal/mol, and the same value is obtained for the KEV ligand. Ruxolitinib shows an identical binding site with a comparable affinity value of −8.3 kcal/mol (Figure 3a–c). Characterizing the binding of ligands and the JAK2 protein, we found that decernotinib demonstrates the highest affinity (−8.7 kcal/mol), while the KEV ligand binds with an affinity of −8.67 kcal/mol, and ruxolitinib with an energy of −8.0 kcal/mol. These structures are shown in Figure 3d–f.

**Figure 4:**
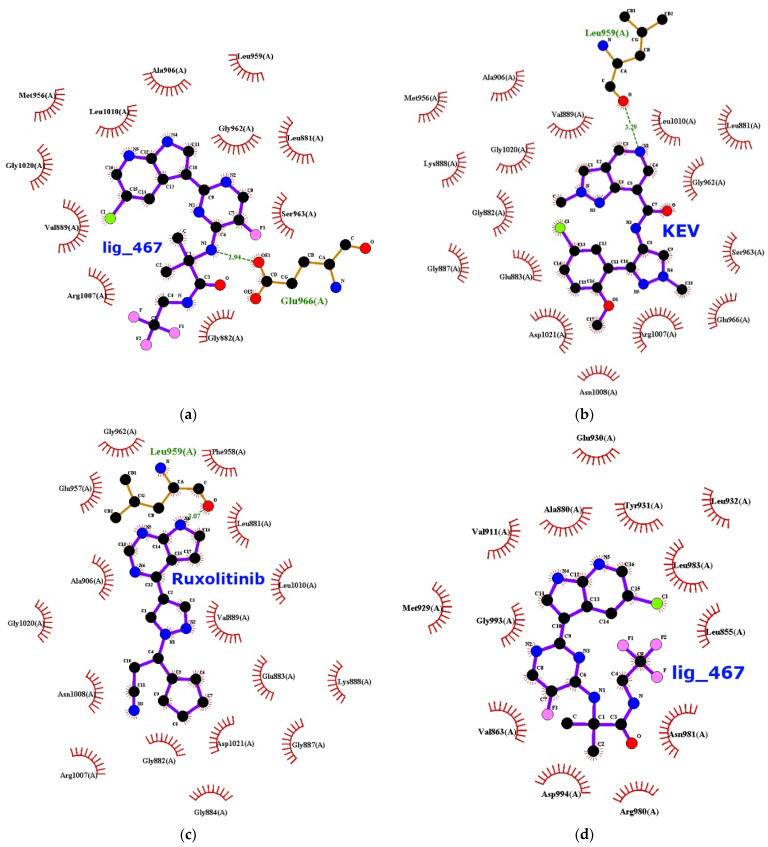

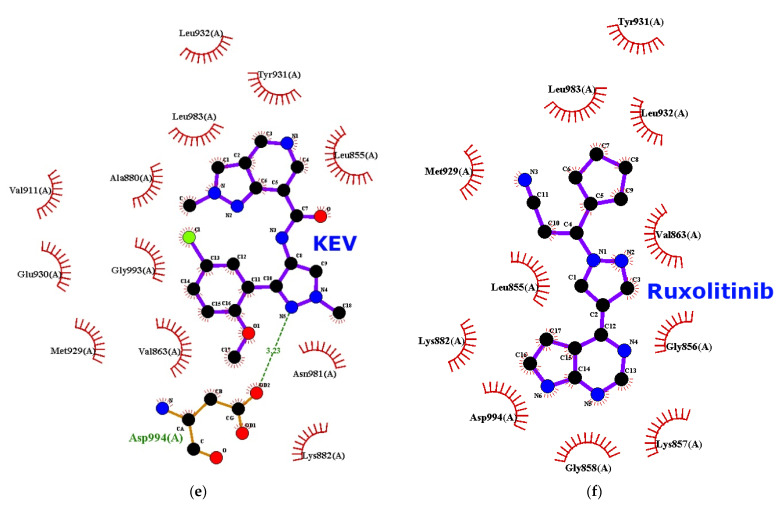
Interactions diagram of JAK1 kinase interactions with decernotinib (**a**), with KEV ligand (**b**), and with ruxolitinib (**c**); JAK2 kinases with decernotinib (**d**), with KEV ligand (**e**), and with ruxolitinib (**f**) (Supplementary materials, Figures S7–S12).

Amino acid residues that are important for binding to the studied ligands are detected using the LIGPLOT+ package, and the results are given below. Initially, during visual analysis of the calculated structures, we assumed that ligands with a pronounced heterocyclic nature, as well as halogen substituents, would be bound by hydrophobic interactions (residues LEU881, GLY884, and GLY962 for JAK1) or hydrogen bonds (ARG879 for JAK1) (Supplementary materials, Table S1). This would achieve a reproducible position of the ligands in the “grotto” of the protein globule. For the JAK2 protein, a similar analysis suggests that the LEU855, GLY858, and LEU983 residues are the source of hydrophobic interactions, whereas the ARG980 residue probably acts as a hydrogen bonding agent. Calculations using the LIGPLOT+ package make it possible to confirm that the main type of interactions that stabilizes the studied complexes are hydrophobic interactions (Figure 4a–c,d–f for JAK1 and JAK2, respectively).

**Figure 5:**
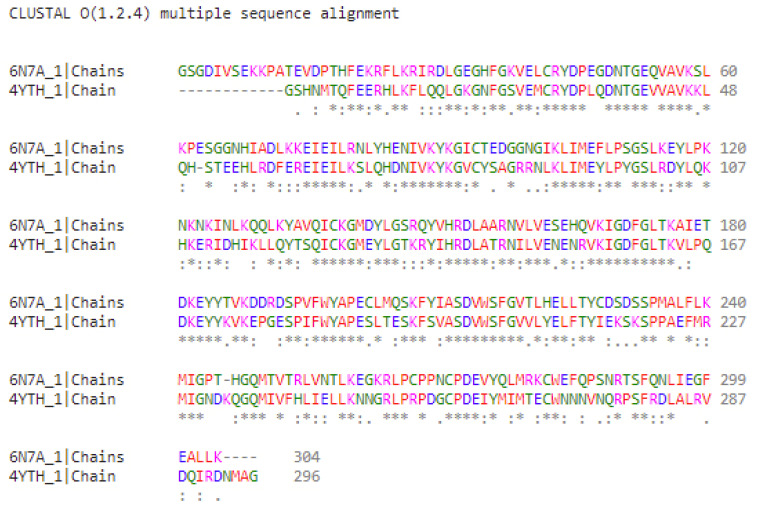
Amino acid sequence alignment (FASTA) results for JAK1 and JAK2 kinases. The symbol (*) under the sequences indicate identity, conservative substitutions (“:”) and semi-conservative substitutions (“.”).

The analysis of the presented data makes it possible to supplement the list of residues responsible for positioning the ligand in the hydrophobic pocket of the JAK1 protein. The hydrogen bond with decernotinib is formed by the GLU966 residue, while the amino acid residue LEU959 interacts with the KEV ligand and ruxolitinib. In JAK2 kinase, LEU855, LEU983, and ASP994 residues are involved in the interactions, while in the case of decernotinib and ruxolitinib ligands, they form only hydrophobic interactions. In the KEV ligand, aspartic acid residue forms a hydrogen bond. When analyzing the structure of these homologous proteins, a question arises about the distribution of amino acid residues over identical sites of the globule, especially over the inner surface of the niche, the “grotto”, where the binding of the studied set of ligands occurs. Therefore, we calculated the alignment of the amino acid sequences of these two kinases (JAK1 and JAK2) using Clustal 0 version 1.2.4 (Conway Institute UCD, Dublin, Ireland) [23]. The results are shown in Figure 5.

It is worth paying attention to the fact that the globule in the PDB file of the JAK1 protein begins with the VAL865 residue and ends with LYS1154 (290 amino acids in total), while, in the JAK2 protein PDB file, it starts with the THR842 residue and ends with MET1130 (289 residues in total). The alignment results show an average homology (approximately 54%) of the amino acid sequences forming the “grotto” of the two isoforms. Despite this, the identified amino acid substitutions are predominantly homologous; for example, the replacement of a hydrophobic amino acid with another hydrophobic one (such as Val–Ile, Ile–Ala, and Leu–Ile), or a negatively or positively charged amino acid with an amino acid of the same charge character (Asp–Glu and Arg–Lys). This determines the high conservatism of the binding of the studied ligands in the hydrophobic pocket of the globule of each of the studied kinases.

## 3. Discussion

The study of the pathological processes molecular basis development in the human body has been a promising area of biomedical research for several decades. Even though often clinical practice, diagnosis, and treatment planning do not require knowledge of the etiology of the disease or pathogenesis, awareness of events at the molecular and cellular level is required. This allows us to propose new approaches for determining risk groups among conditionally healthy people and search for attractive therapeutic targets. The present study contributes to understanding the architecture of ligand binding to JAK family proteins, and contributes to understanding the molecular basis of RA development.

The high conservation of JAK1 and JAK2 amino acid sequences in the kinase activity domain (JH1) determines the structural and physicochemical similarities of most of the targeted kinase inhibitors (ligands). Understanding the molecular basis of ligand and target specificity is important for identifying new drugs and inhibitors of various types of kinases [24]. In the present study, a structural analysis of JAK1 and JAK2 isoform motifs involved in the selective binding of ruxolitinib was performed.

There are few studies focusing on the analysis of the structural features of ruxolitinib binding to kinases. Duan (2014) showed, for the first time, the structure of the active domain pair of chicken c-Src kinase (residues 251-533)-ruxolitinib, at a resolution of 2.26 Å; ruxolitinib has a lower selectivity for c-Src compared to JAK1 [24]. The identity between the amino acid sequences of the kinase domains of JAK1 and c-Src is 34%. The authors observed that the pyrrolopyrimidine rings of ruxolitinib are oriented towards the hinge region. The ligand in the JAK1 cavity is stabilized by two hydrogen bonds with Glu957 and Leu959. Simultaneously, the cyclopentane ring is oriented towards the N-terminus of JAK1, while the propanenitrile group of ruxolitinib interacts with JAK1 [23]. In a recent study by Babu (2022), JAK1 binding selectivity was screened for a set of 52 C-2 methyl/hydroxyethylimidazopyrrolopyridine derivatives, including ruxolitinib [25]. Ruxolitinib forms hydrogen bonds at Leu959 and Glu957 in the JAK1 isoform.

The results obtained in the present study show that the pyrazole derivative ruxolitinib binds with a high affinity to the JAK1 and JAK2 isoforms (Table 2).

The drug ruxolitinib shows similar selectivity for both the JAK isoforms: −8.3 and −8.0 kcal/mol for JAK1 and JAK2, respectively. This result is indirectly confirmed by the literature data (IC50 3.3 ± 1.2 nM for JAK1 and 2.8 ± 1.2 nM for JAK2). The stabilization of the ligands studied in this work, characterized by similar physicochemical properties, was performed primarily by hydrophobic interactions in the JAK “grotto”. In the case of decernotinib and KEV ligands, one hydrogen bond was involved in stabilizing the ligand–target complex.

In our study, we refined the localization of ruxolitinib in the JAK1 and JAK2 “grottoes” with a structural resolution of 1.33 Å and 2.04 Å, respectively. In both the isoforms, ruxolitinib is completely located in the “grotto”. For the JAK1 isoform, the pyrrolopyrimidine and pyrazole rings form a single plane, which is oriented towards the N-terminus of JAK1 and is parallel to the plane of the Leu875–Gly884 β-hairpin in the “grotto” β-barrel. The plane of the cyclopentane ring is approximately perpendicular to the plane of the pyrrolopyrimidine and pyrazole rings, and probably forms a hydrophobic interaction with the Gly884 amino acid residue in the same β-hairpin. The propanenitrile group forms the third plane, which passes through the line of intersection of the first two planes, the plane of the pyrrolopyrimidine ring and the plane of the cyclopentane ring, and is oriented towards the Asn1008–Ile1019 β-hairpin.

In the “grotto” of the JAK2 isoform, the pyrrolopyrimidine and pyrazole rings are located in intersecting planes. The plane of the pyrrolopyrimidine ring, similar to the JAK1 isoform, is oriented towards the N-terminus of JAK2 and is parallel to the plane of the Asn859–Tyr868 β-hairpin in the “grotto” β-barrel. The pyrrolopyrimidine ring forms a single plane with the cyclopentane ring, which is oriented towards the Tyr913–Glu930 β-hairpin. The propanenitrile group forms a third plane oriented towards the Ile982–Ile992 β-hairpin.

For both the JAK isoforms, we observe that the studied complexes are stabilized by hydrophobic interactions. Hydrogen interactions between the ligand and target are not revealed in our study.

## 4. Materials and Methods

### 4.1. Objects of Study

**Figure 6:**
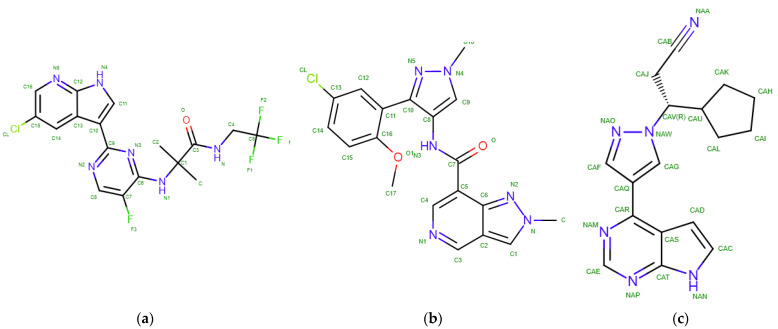
Structural formula of the ligand of (**a**) decernotinib (ligand-467), (**b**) KEV (ligand is drug 39 from), (**c**) ruxolitinib.

Flexible molecular docking was performed for the ligands: (a) decernotinib (PDB ID 4YTH), (b) KEV with the chemical name N-[3-(5-chloro-2-methoxyphenyl)-1-methyl-1H-pyrazol-4-yl]-2-methyl-2H-pyrazolo [4,3-c]pyridine-7-carboxamide (PDB ID 6N7A) [26], and (c) ruxolitinib, a selective antitumor inhibitor of JAK kinase (Figure 6).

Decernotinib (VX-509) is a JAK3 inhibitor (Table 3). This experimental drug is characterized by high selectivity for the JAK3 isoform.

The main objective of our study was to identify the ruxolitinib ligand, an antitumor agent and a selective inhibitor of the JAK1 and JAK2 isoforms. These kinases facilitate the signaling of numerous cytokines and growth factors that play an important role in hematopoiesis and immune system function (Table 3).

As shown in Table 3, the physicochemical properties of the ligands are similar.

Two isoforms were chosen as targets for modeling: JAK1 (PDB ID 6N7A) and JAK2 (PDB ID 4YTH) (Table 4).

### 4.2. Molecular Docking

Data preparation for docking calculations was carried out according to standard protocols [27,28,29]. Ligand–target binding and binding energy rankings were performed using the AutoDock VINA 1.1.2 software package [30]. The results were visualized using AutoDockTools 1.5.6. RC3 [18]. The projection schemes of the ligand–receptor interactions were drawn using LIGPLOT 1.3.6 [28].

The spatial structures of the target protein models obtained from PDB were purified from the solvent, ligand, and buffer molecules. In the MGLTools package (CCSB, San Diego, CA, USA), charges were placed on the proteins, which made them a receptor, a target for searching for optimal binding sites of the studied ligands [31]. The structures of the ligands were created using the HyperChem molecular constructor (http://www.hypercubeusa.com/) and sequentially optimized, first by using the Assisted Model Building with Energy Refinement (AMBER) force field [32], and then quantum-chemically optimized using parametric method 3 (PM3) [33]. The arrangement of charges on the ligand molecule and its protonation/deprotonation were carried out automatically using the MGLTools 1.5.6 package [31]. The calculations were performed on a server using IntelXEON processors (40 cores).

The task of analyzing the docking results was significant. The LIGPLOT+ package was used to obtain “sweeps” of interactions [34]. From the three-dimensional coordinates of the atoms of the protein–ligand complex, interaction diagrams were generated that depict the schemes of hydrogen bonds, as well as hydrophobic contacts between the ligand and elements of the main or side chain of the protein.

## 5. Conclusions

Molecular modeling data shows that the drug ruxolitinib selectively binds the JAK1 and JAK2 isoforms with a binding affinity of −8.3 and −8.0 kcal/mol, respectively. In both the JAK isoforms, ruxolitinib resides entirely in the “grotto” or binding cavity. The stabilization of the ligand in the “grotto” of kinases is performed primarily by hydrophobic interactions, including 18 JAK1–ligand contacts and 11 JAK2–ligand contacts. For the first time, we detail the amino acid residues of the JH1 domains of protein globules, which are responsible for the correct positioning of the ligand and its retention. Agr879, Leu881, Gly884, and Gly962 are involved in the formation of the JAK1–ligand complex; hydrophobic amino acid residues Leu855, Gly858, Arg980, and Leu983 are also involved in the formation of the JAK2–ligand complex.

## Figures and Tables

**Table 1:** Structural and physicochemical properties of “grottoes” for JAK1 and JAK2.

“Grotto”	SASA, Å^2^	HB *	Nonpolar a.a. (%)	Polar a.a. (%)	Charged a.a. (%)	Nonpolar “Cross-Elements” Contacts	Nonpolar “Inside-Elements” Contacts	Distance ** of “Cross-Elements” Contacts (Å ± SD)	Distance of “Inside-Elements” Contacts (Å ± SD)
JAK1 (865–1154)	29,255.336	189	36.8	30.9	31.6	310	43	8.28 ± 1.37	7.80 ± 1.54
JAK2 (842-1130)	31,252.359	198	38.4	30.4	30.4	333	38	8.25 ± 1.40	7.67 ± 1.62

HB *—number of hydrogen bonds; distance **—between nonpolar a.a.

**Table 2:** Steric and energetic parameters of kinase binding to ligands.

Ligand	JAK1 (6N7A), kcal/mol	Locus of “Grotto”	HB *	Hydrophobic Bonds **	JAK2 (4YTH), kcal/mol	Locus of “Grotto”	HB	Hydrophobic Bonds
Decernotinib	−8.7	ARG879, LEU881, GLY884, GLY962	1	10	−8.7	LEU855, GLY858, ARG980, LEU983	0	13
Ligand KEV	−8.7	ARG879, LEU881, GLY884, GLY962	1	15	−8.6	LEU855, GLY858, ARG980, LEU983	1	12
Ruxolitinib	**−8.3**	ARG879, LEU881, GLY884, GLY962	**0**	**16**	**−8.0**	LEU855, GLY858, ARG980, LEU983	0	11

*—number of hydrogen bonds between the target and the ligand; **—number of hydrophobic bonds with distances between the contacting amino acid residues of the target and the ligand less than 10 Å.

**Table 3:** Physicochemical properties of the ligands used in the work.

Ligand	IUPAC (Formula)	Class	Mw, Da	Donor HB *	Acceptor HB	TPSA **, Å^2^	SC ^3^*	Selectivity	IC50, Nm ^4^*
Decernotinib	(2R)-2-methyl-2-[[2-(1H-pyrrolo [2,3-b]pyridin-3-yl)pyrimidin-4-yl]amino]-N-(2,2,2-trifluoroethyl)butanamide	Pyrimidine	392.4	3	8	95.6	0	JAK3	2.5 ±0.7
KEV	N-[3-(5-chloro-2-methoxyphenyl)-1-methyl-1H-pyrazol-4-yl]-2-methyl-2H-pyrazolo [4,3-c]pyridine-7-carboxamide(C19H17ClN6O2)	Pyrazole	396.8	1	5	86.9	0	JAK1	N/A
Ruxolitinib	(3R)-3-cyclopentyl-3-[4-(7H-pyrrolo [2,3-d]pyrimidin-4-yl)pyrazol-1-yl]propanenitrile	Pyrazole	306.4	1	4	83.2	1	JAK1 and JAK2	3.3 ± 1.2 (JAK1)2.8 ± 1.2 (JAK2)

*—hydrogen bond; **—topological polar surface area; ^3^*—stereocenter; ^4^*—cell-free assay.

**Table 4:** Structures of JAK1 and JAK2 proteins used for redocking with ruxolitinib.

Target	PDB ID	Uniprot ID	Resolution, Å	Ligand	Method	Sequence Length, a.a.	Native Ligand	Binding Affinity with Native Ligand, nM
JAK1	6N7A	P23458	1.33	Ruxolitinib	X-ray	304	KEV	4.3
JAK2	4YTH	O60674	2.04	Ruxolitinib	X-ray	296	Decernotinib	2

## Data Availability

Not applicable.

## Supplementary Materials

The supporting information can be downloaded at: https://www.mdpi.com/article/10.3390/ijms231810466/s1.
