# Supplementary material for: Atomic Simulation of the Binding of JAK1 and JAK2 with the Selective Inhibitor Ruxolitinib
Source: Int J Mol Sci. 2022 Sep 9;23(18):10466. doi: 10.3390/ijms231810466 (PMC9504736; doi:10.3390/ijms231810466)

Supplementary Materials

Figures

Figure S1. JAK1: ligand binding site with decernotinib

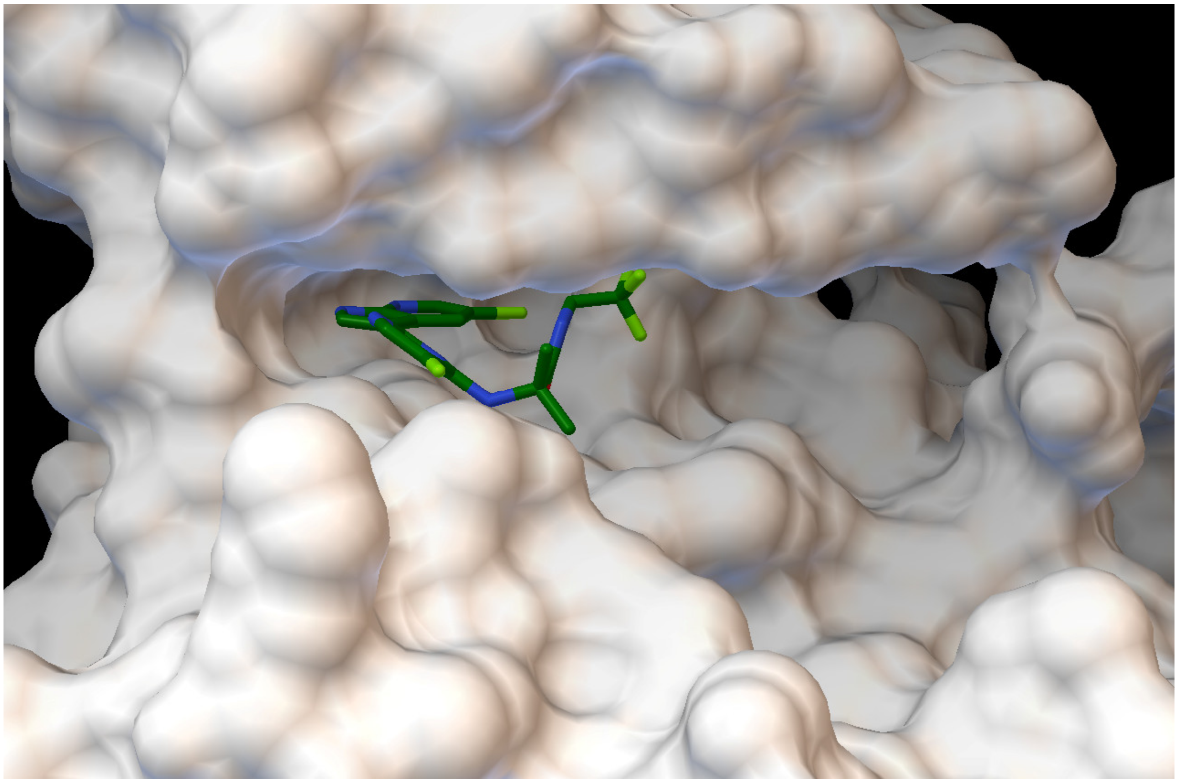

Figure S2. JAK1: ligand binding site with KEV

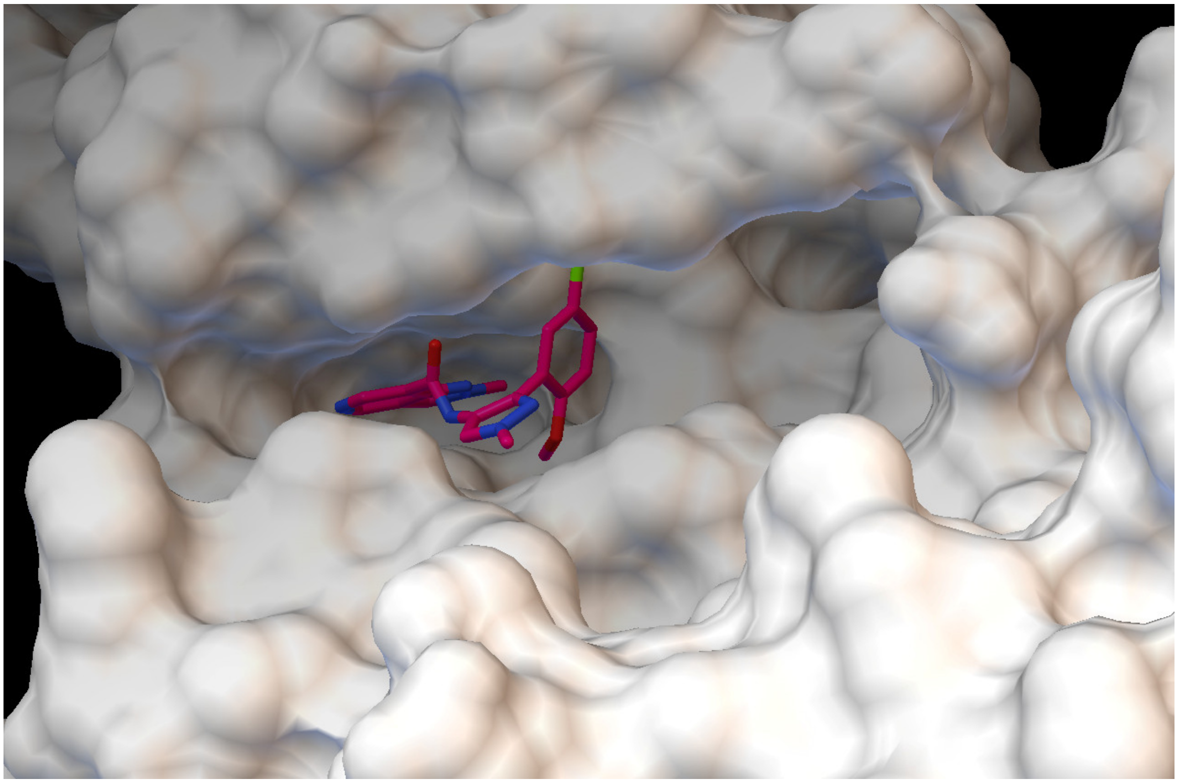

Figure S3. JAK1: ligand binding site with ruxolitinib

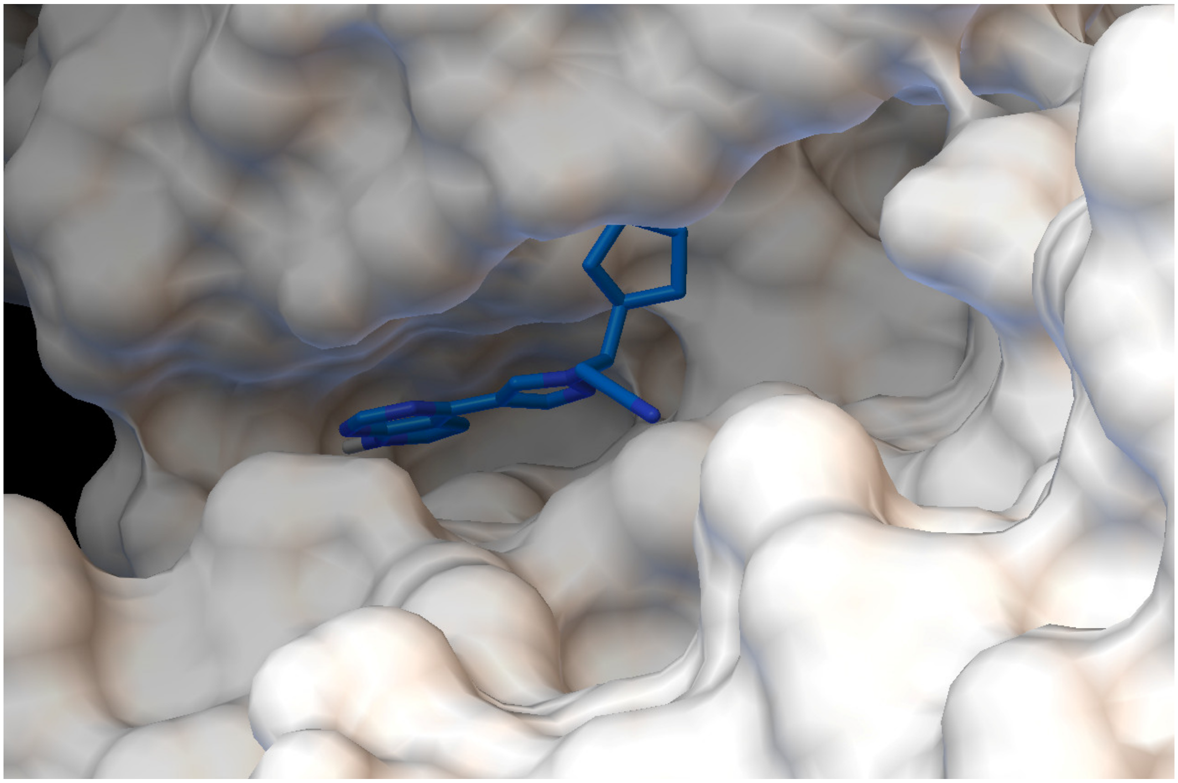

Figure S4. JAK2: ligand binding site with decernotinib

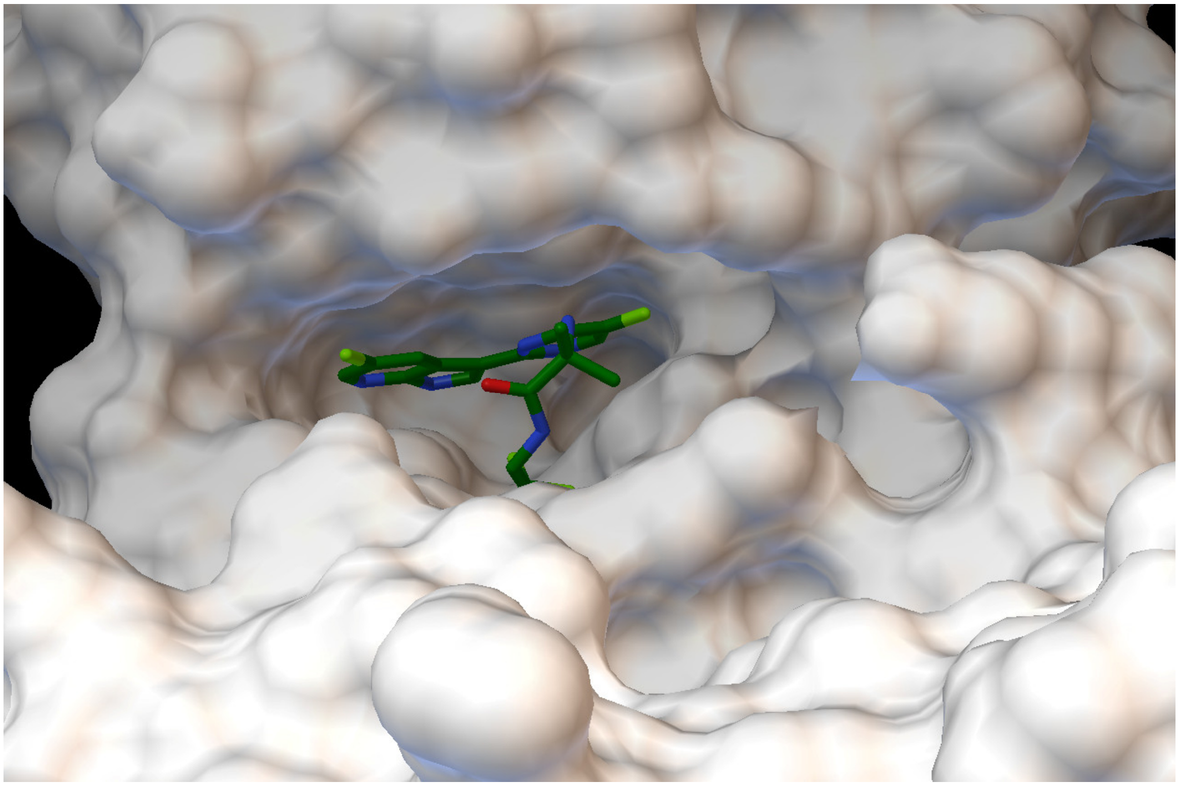

Figure S5. JAK2: ligand binding site with KEV

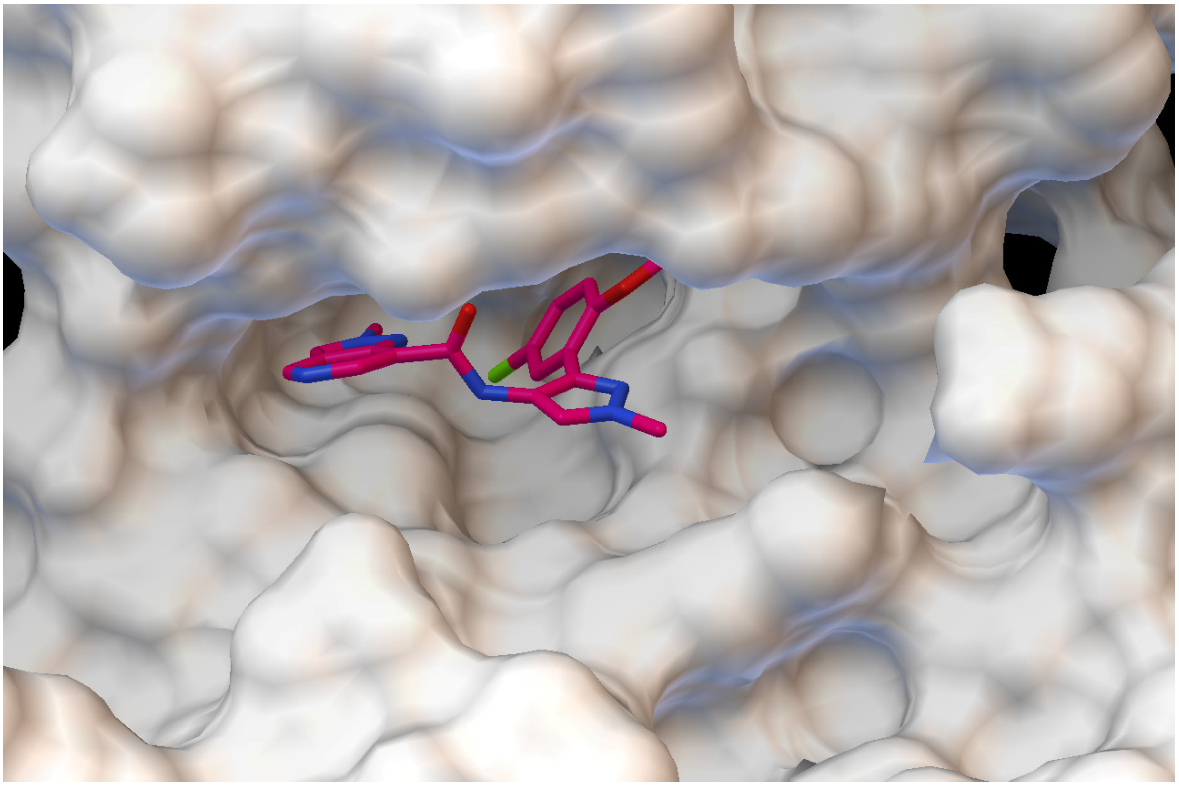

Figure S6. JAK2: ligand binding site with ruxolitinib

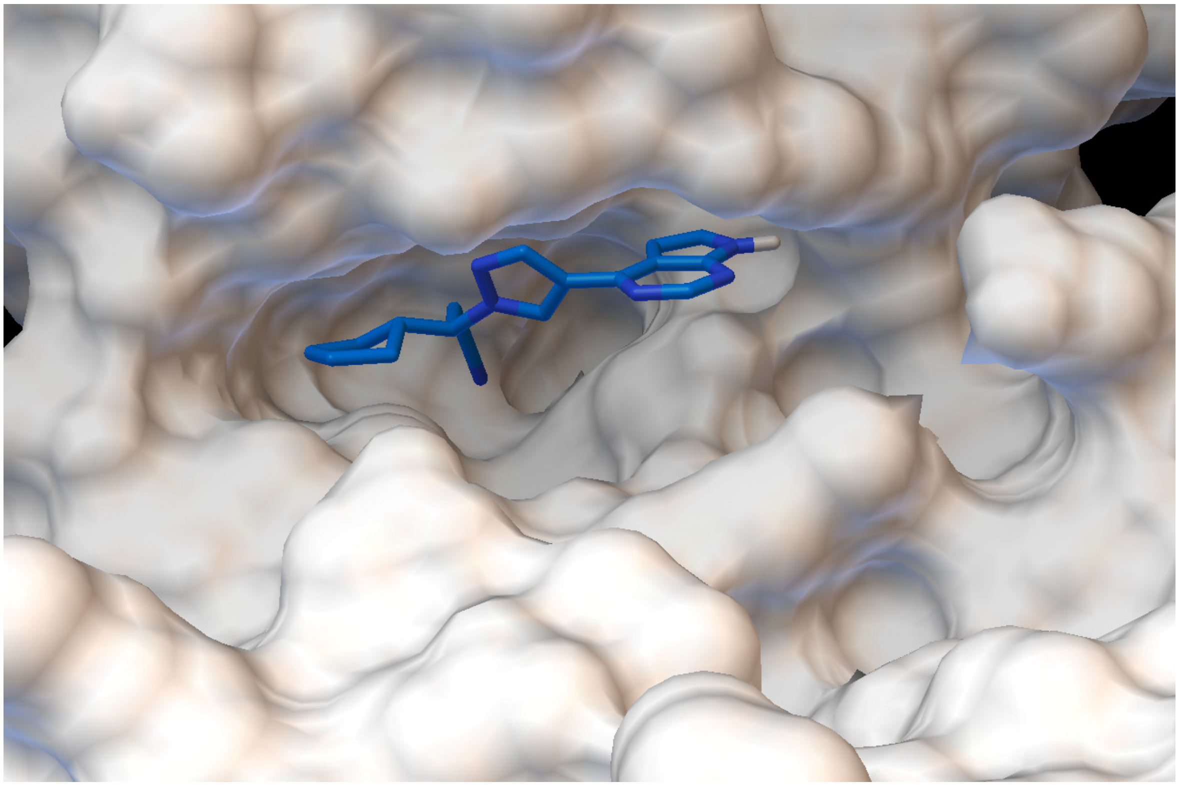

Figure S7. Interactions diagram of JAK1 kinase interactions with decernotinib

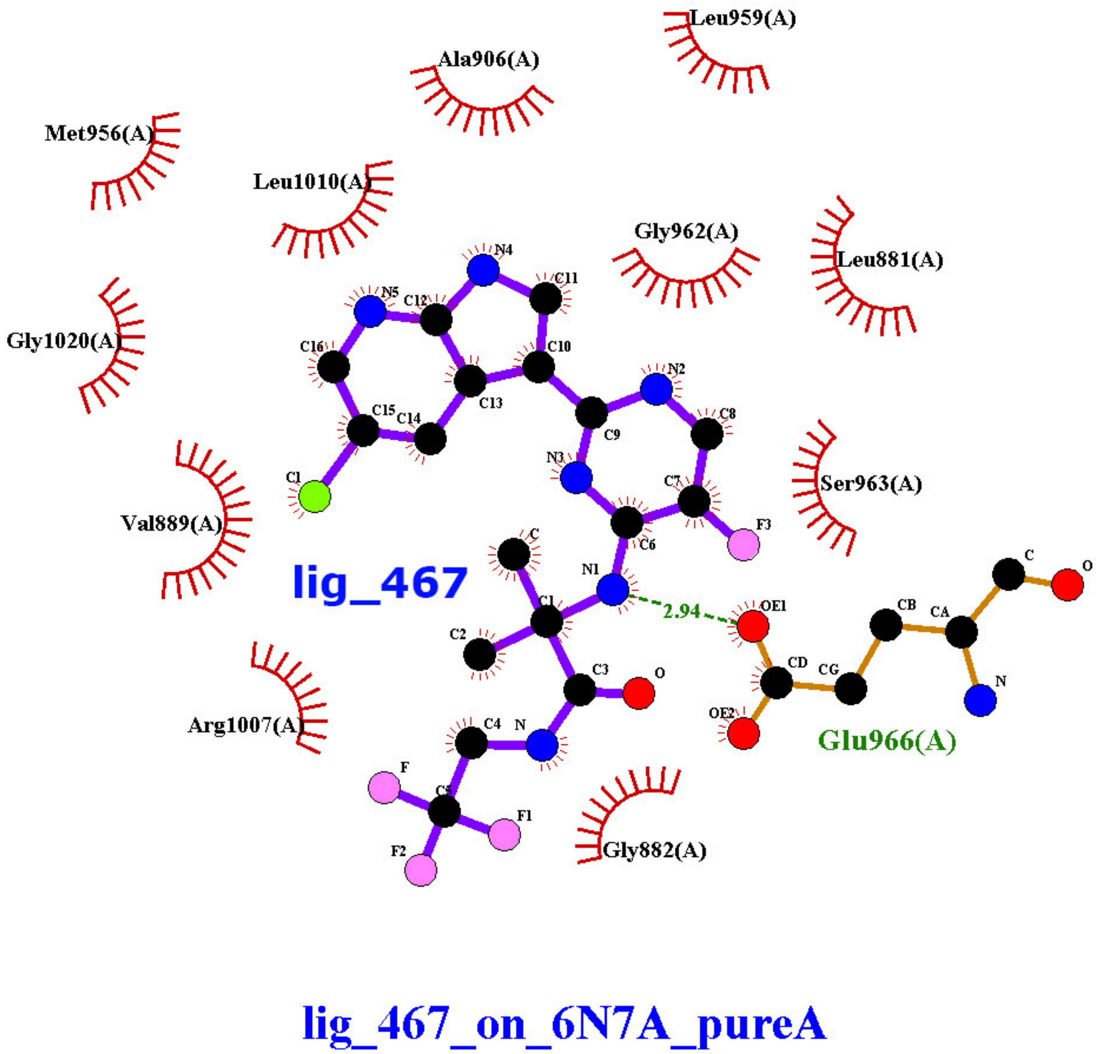

Figure S8. Interactions diagram of JAK1 kinase interactions with KEV

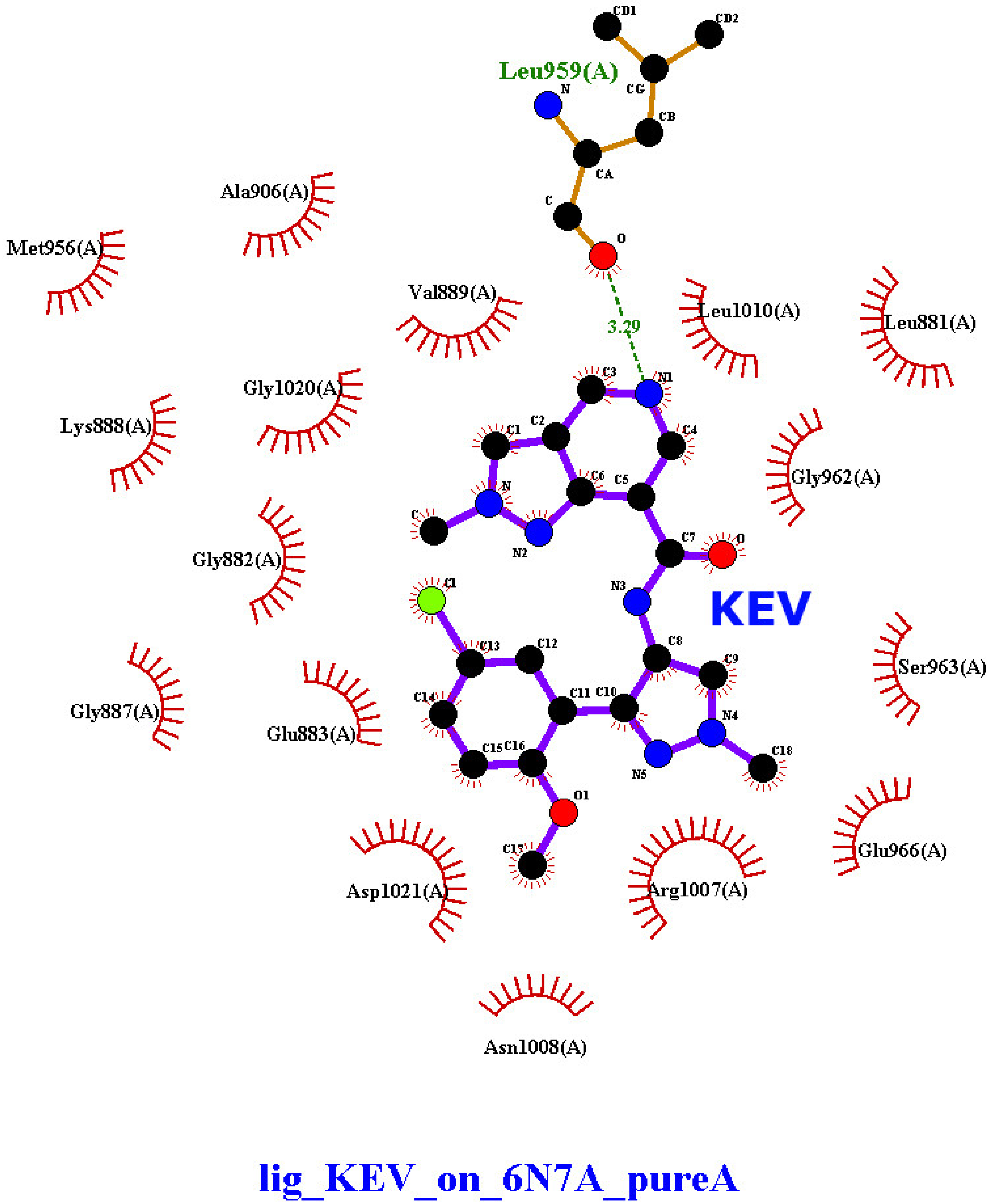

Figure S9. Interactions diagram of JAK1 kinase interactions with ruxolitinib

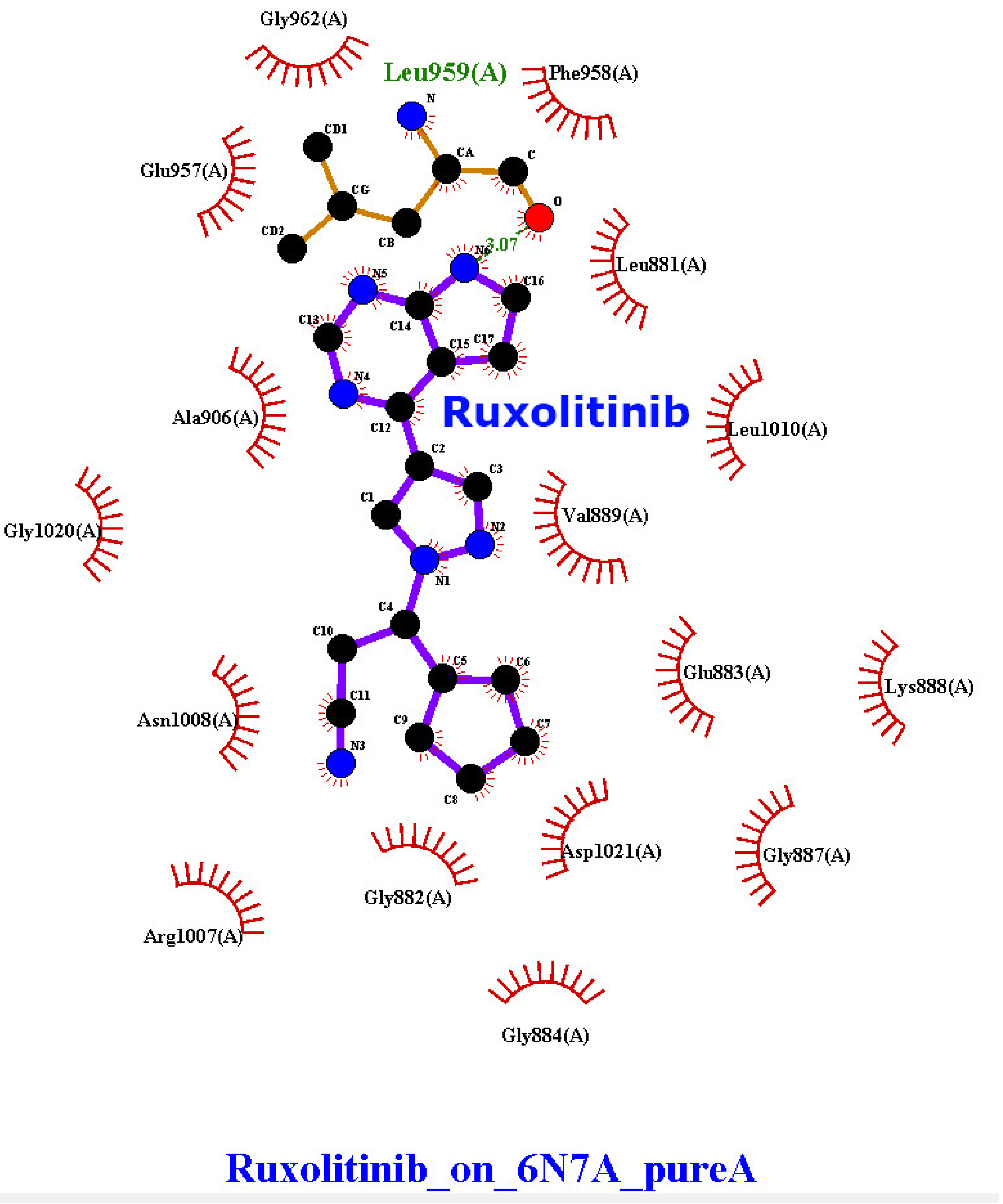

Figure S10. Interactions diagram of JAK2 kinases with decernotinib

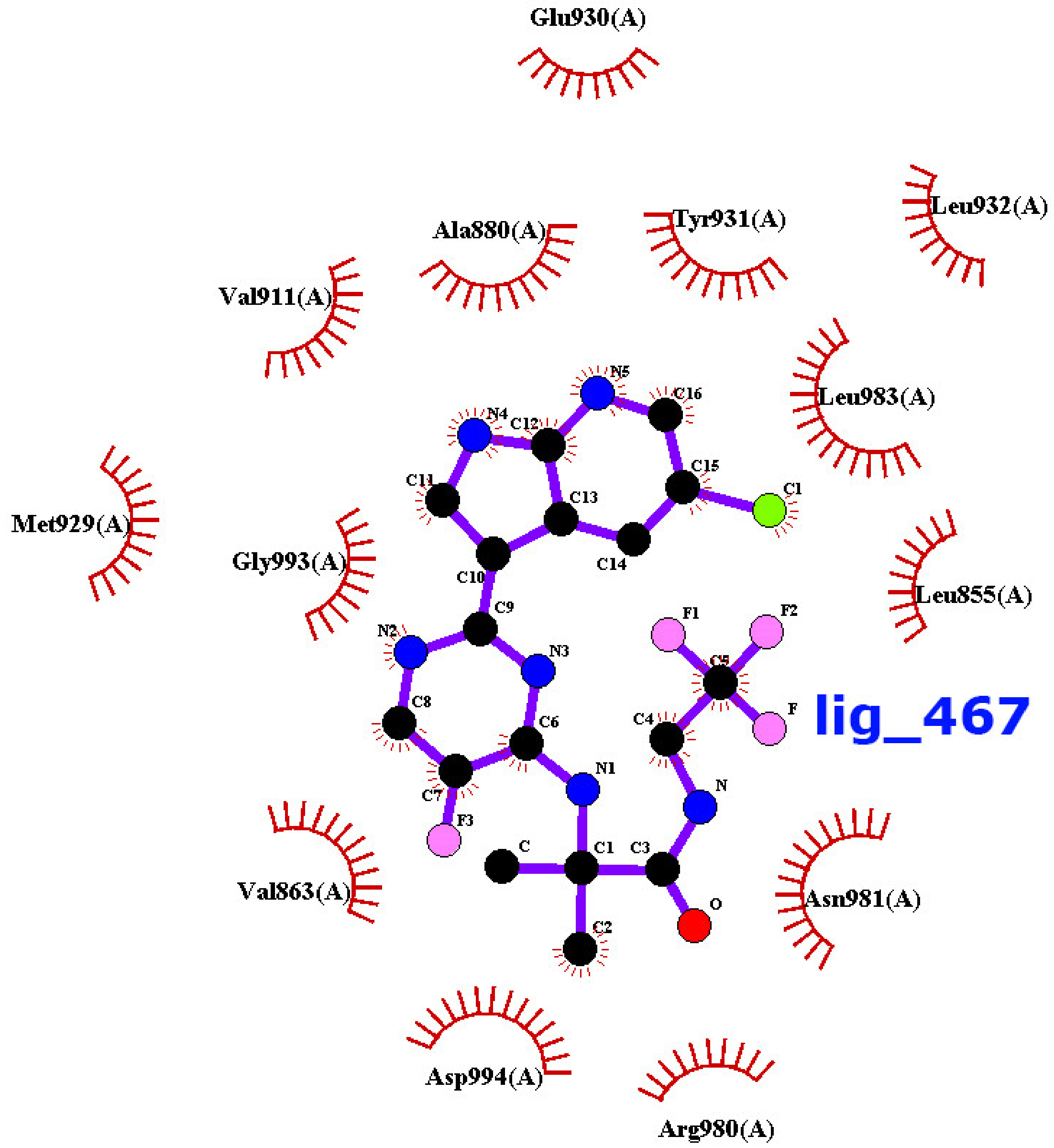

lig_467_on_4YTH_pureA

Figure S11. Interactions diagram of JAK2 kinases with KEV ligand

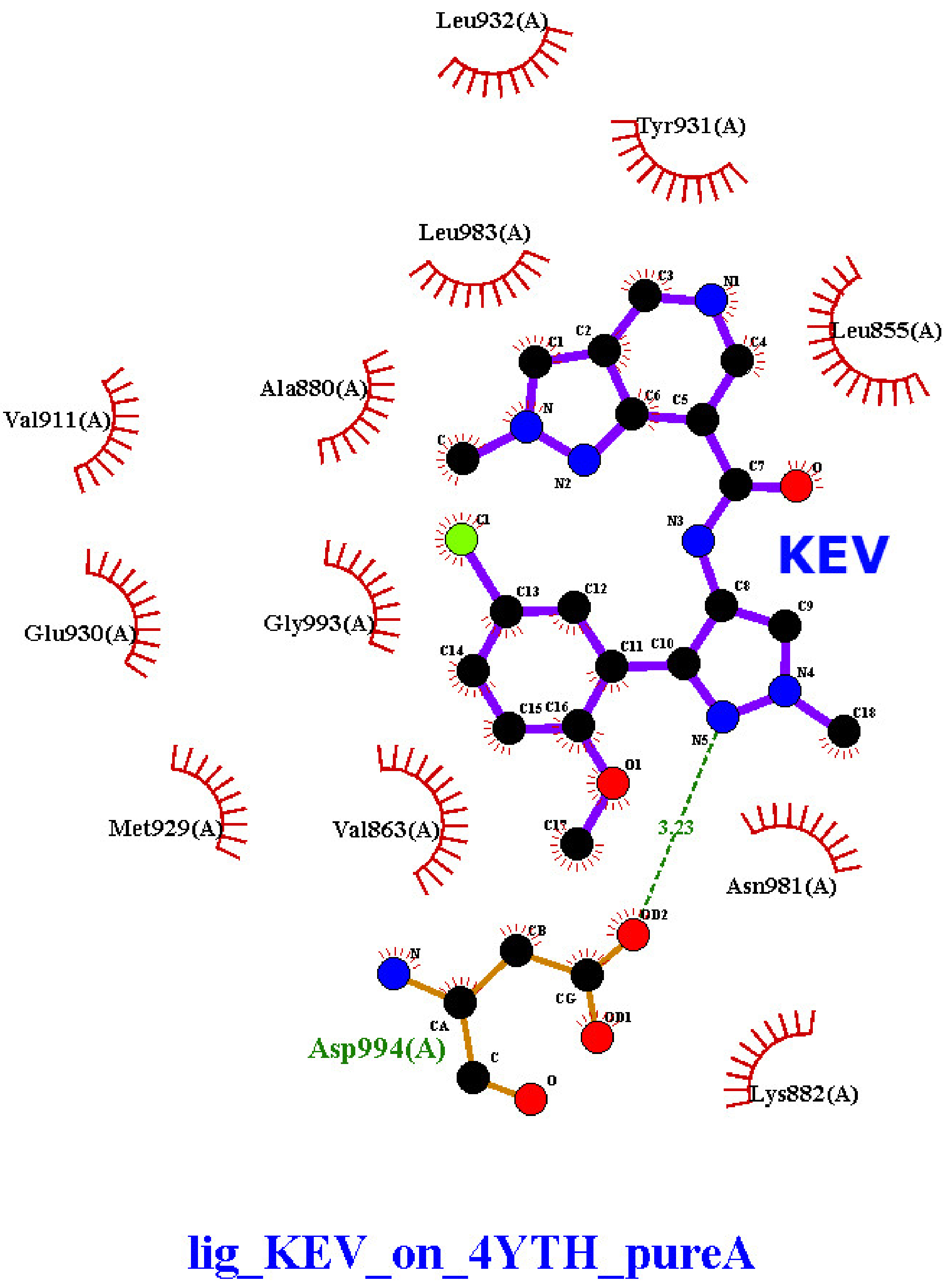

Figure S12. Interactions diagram of JAK2 kinases with ruxolitinib

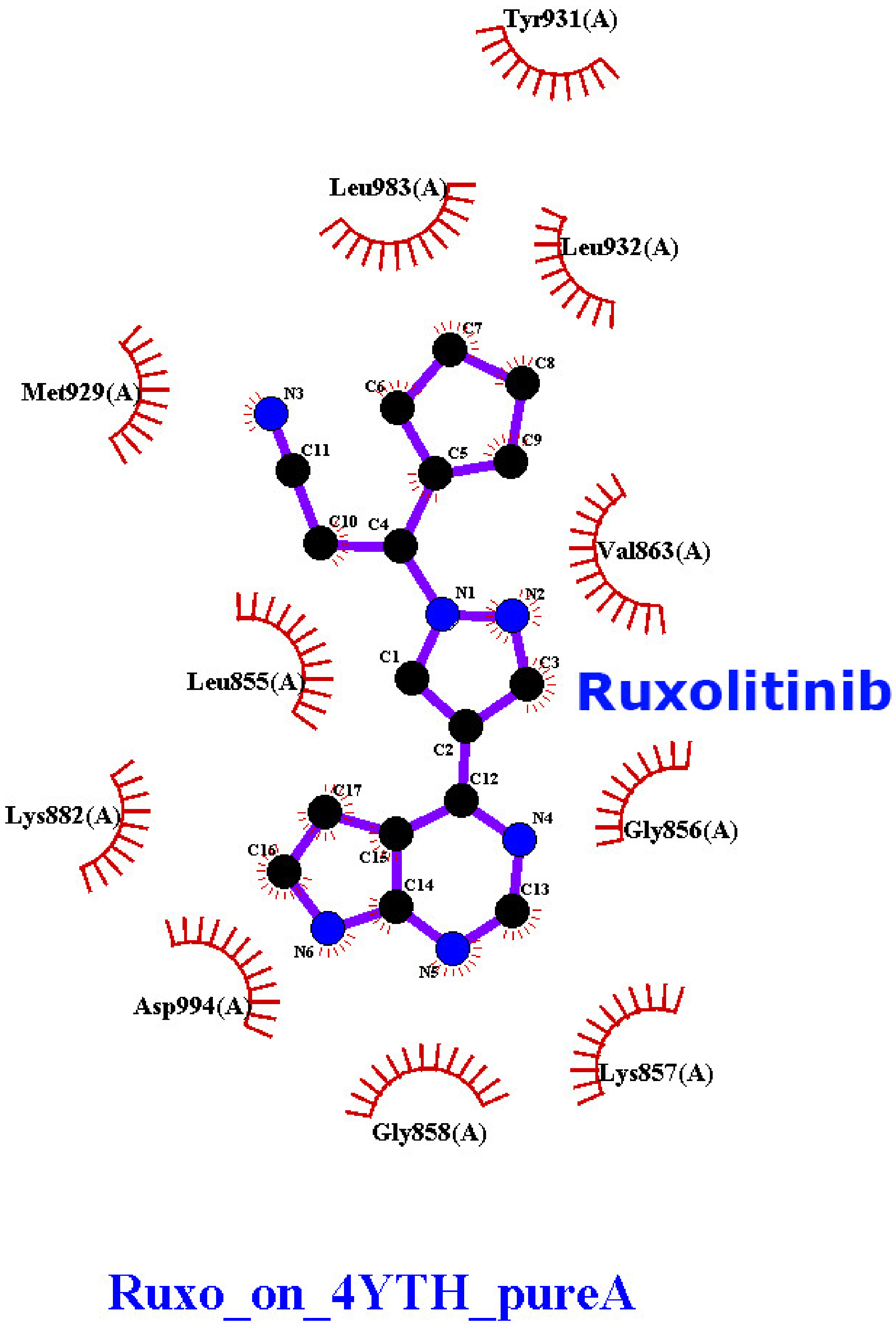

Supplement: Supplementary file 1 [file ijms-23-10466-s001.zip › ijms-1889007-supplementary.pdf]
